# Meaning of Ambiguity: A Japanese Survey on Synthetic Biology and Genome Editing

**DOI:** 10.3389/fsoc.2019.00081

**Published:** 2019-12-17

**Authors:** Aiko Hibino, Go Yoshizawa, Jusaku Minari

**Affiliations:** ^1^Faculty of Humanities and Social Sciences, Hirosaki University, Hirosaki, Japan; ^2^Work Research Institute (AFI), OsloMet – Oslo Metropolitan University, Oslo, Norway; ^3^Uehiro Research Division for iPS Cell Ethics, Center for iPS Cell Research and Application (CiRA), Kyoto University, Kyoto, Japan

**Keywords:** public engagement, public perception, synthetic biology, genome editing, self-image, future generations, intermediate response, ambiguity

## Abstract

Synthetic biology and genome editing have become increasingly controversial issues, necessitating careful attention and engagement with the public. Our study examined ambiguity in public perception about emerging biotechnologies through the use of several intermediate response options in a survey. To understand the relationship between respondents' thoughts and attitudes, we also examined how respondents' indecision is related to their cognitive concept of “self” as well as their interpretation of “future generations.” An online survey of 994 respondents living in Japan revealed that around 80% hold intermediate attitudes (two-sided, non-judgmental, or reserved attitudes) toward synthetic biology and genome editing. These results revealed that respondents who have a narrow self-concept tend to postpone decisions about the application of emerging technologies. In contrast, those with a broad self-concept tend to adopt an ambivalent attitude and are more short-sighted, but make judgments based on the impact of their decisions on current and future generations. This study thus demonstrates that public views are more diverse and nuanced than those obtained from conventional public surveys for policy making.

## Introduction

Biotechnologies associated with genomic data are dramatically advancing. These advances have provided numerous benefits to society, especially in the areas of medicine, energy, and food production. However, as emerging biotechnologies have a broad influence on many facets of society, it has become more important to assess the social implications by conducting research to identify public attitudes regarding the early phase technology development (Guston and Sarewitz, 2002). Despite the current established regulations and agreements, certain ethical, legal, and social issues need to be addressed to reflect regional, national, and global contexts (Yoshizawa et al., 2014, 2017; Minari et al., 2018). Particularly, it is controversial how to practically apply germline genome editing for humans and the natural environment. One of the latter applications is now called “gene drives” as it can rapidly disseminate specific genetic properties through a population (Caplan et al., 2015; Carroll and Charo, 2015; Lander et al., 2019). The convergence of genome editing technologies and synthetic biology has yielded rapid advancement in genome synthesis technologies (Boeke et al., 2016; Chari and Church, 2017).

Whereas many scholars have already provided significant insight into public views on the use of genome editing and synthetic biology (McCaughey et al., 2016; Gaskell et al., 2017; Rose et al., 2017; Scheufele et al., 2017; Weisberg et al., 2017; Uchiyama et al., 2018), relatively few studies have conducted in-depth analysis of the contingent nature of public views on emerging technologies (Dietrich and Schibeci, 2003). Since most of their impact lies in the future, a defining attribute of emerging technologies is *ambiguity* (Rotolo et al., 2015).

Ambiguity has been examined in previous studies on public understanding of science, one of which analyzes the meaning of “don't know” (DK) responses to surveys (Bauer and Joffe, 1996). Earlier studies have clarified several types of DKs; for example, “alienated DK” and “ambivalent DK” were explored through quantitative analysis (Hibino, 2010), and the associated ignorance was classified as either a “division of labor” or “deliberate choice” (Turner and Michael, 1996). While such analyses are useful for interpreting the meaning of intermediate answers, they attempt to illuminate the social reasons for DK responses. Respondents in Japan in particular, as well as in several other neighboring countries, tend to provide mid-point responses (Chen et al., 1995; Johnson et al., 2005; Tasaki and Shin, 2017). While policy makers must make decisions in complex social systems by considering a range of technical, political, moral, and ethical concerns (Sanderson, 2006), it is also significant to examine public ambiguous responses as usable evidence for policy making and global governance of emerging technologies.

The purpose of our study is to explicate ambiguity in public perception regarding synthetic biology and genome editing. For this purpose, we developed a novel questionnaire consisting of more intermediate response options than “neutral” or “don't know.” Thereafter, we explored how respondents' indecision is relevant to their cognitive concept of “self” and “future generations.”

## Methods

In December 2017, we conducted an online survey of 994 respondents (male = 529, female = 465) through a survey research company based in Japan. Respondents were Japanese, aged between 16 and 60 (mean = 37.0; SD = 12.41), and were required to be living in Japan during the survey. Gender was approximately balanced across the five age groups. Table 1 shows the demographic variables of gender, age, education, and employment, as well as the awareness rate of “synthetic biology” and “genome editing.” While the survey includes comprehensive questions related to knowledge of emerging biotechnologies and views of nature, our focus is on the following four areas:

1. Attitude toward synthetic biology: “Do you agree with the development of synthetic biology?” (5 categories: “agree”; “disagree”; “I agree and disagree”; “I do not think I can make a judgement on my own”; and “I cannot judge at this time”). A brief description of synthetic biology was given to respondents before their response; “There is increasing attention on synthetic biology that attempts to elucidate the origin and essence of life by creating artificial cells.”
2. Attitude toward genome editing: “Do you agree with the development of genome editing?” (5 categories: “agree”; “disagree”; “I agree and disagree”; “I do not think I can make a judgement on my own”; and “I cannot judge at this time”) A brief description of genome editing was given to respondents before their answering; “There is increasing attention on genome editing that enables the modification of genes in a more accurate and effective manner than previous technologies.”
3. The cognitive image of the self: This study asked respondents, “which items do you find relatable as a part of yourself?” and provided multiple choices (“hair,” “limbs,” “spirit,” “age,” “my products,” “the status,” “moral,” “friend,” “school or company,” “earth,” “nature,” “other,” and “none of them corresponds to my choice”), by referring to Ikeuchi et al. (1999). The total amounts of the number of chosen items except “other” and “none of them” were used as an index of the broadness of the cognitive image of the self (from 0 to 11; mean = 2.8). Categories were set by referring to the scale of the *extended self* that was originally proposed by Prelinger (1959), modified by Ikeuchi et al. (1999) and has been examined mainly in psychological researches (Belk, 2013).
4. The cognitive image of future generations: “Which category do you mean by future generations?” (6 categories: “Those younger than me”; “Those younger than primary school students”; “Those who will be born in the next few years”; “Those who will live several decades in the future”; “Those who will live a thousand years later”; and “All people in the future”).

**Table 1:** Demographic responses and knowledge of emerging biotechnologies in the current sample (*N* = 994).

**Question/response options**	**% of sample**
**Gender**	
Male	53.2
Female	46.8
**Age**	
16–19	12.5
20–29	20.6
30–39	24.7
40–49	23.9
50–60	18.2
**Education**	
Junior high school	7.9
Completed high school	28.3
College/undergraduate/postgraduate degree	63.2
Other	0.6
**Employment status**	
Employees	57.5
Self-employed	6.9
Unemployed	33.1
Unknown	2.4
**Have heard of synthetic biology**	
Yes	9.1
No	90.9
**Have heard of genome editing**	
Yes	32.0
No	68.0

Relationships between items were examined and a categorical data analysis was performed using Akaike Information Criterion (AIC) (Sakamoto, 1992). The distribution dependency of a specified variable (response variable) on other variables (explanatory variables) was derived and evaluated using AIC.

The questionnaire includes the following items; gender, age, residential areas, employment status, educational background, interests related to topics of emerging technologies, awareness of synthetic biology/genome editing, attitude toward synthetic biology/genome editing, important aspects of applications of synthetic biology/genome editing, comfort activities in daily lives, discomfort activities in daily lives, cognitive concept of the self, and cognitive concept of future generations.

Categorical Data Analysis Program Package was used to evaluate dependencies of the response variables, attitudes toward synthetic biology and genome editing, and variables related to cognitive self-concept and future generations. The same analysis was also applied to clarify the proper division pattern of explanatory variables. CATDAP-02 searched for optimal categorization of continuous values for cognitive self-concept and future generations.

## Results

### Intermediate Attitudes Toward Emerging Biotechnologies

For this study, three intermediate options were prepared to determine specific perceptions of respondents. Table 2 shows the distribution of public attitudes toward synthetic biology and genome editing. In total, 78.9% respondents chose responses indicating intermediate attitudes (two-sided, non-judgmental, and reserved) toward synthetic biology. 31.0% answered “I agree and disagree” (two-sided); 17.6% answered “I don't think I can make a judgment on my own” (non-judgmental); and 30.3% answered “I don't think I can make a judgment at this time” (reserved). The two-sided and reserved options were dominant among intermediate answers, while only 21.1% of respondents explicitly stated affirmative or negative opinions. 17.1% of respondents chose “agree” for a question asking about the necessity of technology development while 4.0% chose “disagree.”

**Table 2:** Responses to the survey question, “Do you agree with the development of emerging biotechnology?” (*N* = 994).

	**Synthetic biology (%)**	**Genome editing (%)**
Agree	17.1	10.5
Disagree	4.0	7.4
I agree and disagree (two-sided)	31.0	30.2
I don't think I can make a judgement on my own (non-judgmental)	17.6	15.8
I cannot judge at this time (reserved)	30.3	36.1

The response distribution for genome editing was quite similar to that for synthetic biology (Table 2). Intermediate options were also dominant, with 30.2% of respondents choosing the two-sided response, 15.8% choosing non-judgmental, and 36.1% choosing reserved.

### Explanatory Variables for Attitudes Toward Emerging Biotechnologies

#### Determinants of Attitudes

The distribution dependency of attitudes toward synthetic biology on other variables was evaluated. The highest variables were cognitive image of the self (AIC = −106.40), awareness of genome editing (AIC = −75.57), cognitive image of future generations (AIC = −40.30), and awareness of synthetic biology (AIC = −34.23).

#### Role of Cognitive Concept of the Self

The cognitive concept of the self had a relatively strong association with attitude toward emerging biotechnologies. Those with a broad cognitive self-concept, who perceived many components of the world as being relatable to humans, were more likely to choose “agree” (25.9%) or have a two-sided attitude (40.3%) toward synthetic biology (Table 3). On the contrary, those with a narrow cognitive self-concept, who perceived few components as being relatable to humans, tended to show a reserved attitude (middle = 25.6%, narrow = 61.1%). These findings suggest that individuals with a narrower concept of the self are more likely to postpone judgment on emerging biotechnologies.

**Table 3:** Cognitive self-concept and attitudes toward emerging biotechnologies.

	**Agree**	**Disagree**	**Two-sided**	**Non-judgmental**	**Reserved**	**Sum**	***N***
**SYNTHETIC BIOLOGY (%)**							
Narrow (0)	5.3	3.2	20.0	10.5	61.1	100.0	190
Middle (1 to 5)	18.6	4.4	32.2	19.2	25.6	100.0	665
Broad (6 to 11)	25.9	3.6	40.3	19.4	10.8	100.0	139
**GENOME EDITING (%)**							
Narrow (0)	3.7	3.7	16.3	13.7	62.6	100.0	190
Middle (1 to 6)	12.0	7.4	32.3	17.3	31.1	100.0	718
Broad (7 to 11)	12.8	16.3	43.0	8.1	19.8	100.0	86

The attitude distribution for genome editing resembled that for synthetic biology. Those with a narrow cognitive self-concept tended to postpone judgement on genome editing (Table 3). However, those with a broad cognitive self-concept followed different response patterns between synthetic biology and genome editing. Compared to the attitude toward synthetic biology, respondents with a broad self-concept expressed greater disagreement and had more reserved attitudes toward genome editing.

#### Role of Cognitive Concept of Future Generations

The cognitive concept of future generations also had an association with the attitude toward emerging biotechnologies. The original statements related to the cognitive concept of future generations consisted of six categories: (1) “Those younger than me,” (2) “Those younger than primary school students,” (3) “Those who will be born in the next few years,” (4) “Those who will live several decades in the future,” (5) “Those who will live a thousand years later,” and (6) “All people in the future.” Categorical Data Analysis Program Package (CATDAP-02) reclassified these six categories into two—near future (categories 1 to 4) and distant future (categories 5 to 6). The explanatory powers of the two categories (synthetic biology: AIC = −40.30; genome editing: AIC = −33.14) were stronger than those of the original six categories (synthetic biology: AIC = −23.62; genome editing: AIC = −12.24).

Based on the above analysis, Table 4 illustrates that attitudes toward emerging biotechnologies are associated with the image of future generations. For synthetic biology, respondents who considered future generations as being in the near future tended to have affirmative (19.0%) and two-sided (34.4%) attitudes. On the contrary, respondents who considered future generations as being in the distant future had strong tendencies to reserve their attitude (45.8%). The response pattern for genome editing bore a strong resemblance to that seen for synthetic biology (Table 4).

**Table 4:** Cognitive concept of future generations and attitudes toward emerging biotechnologies.

	**Agree**	**Disagree**	**Two-sided**	**Non-judgmental**	**Reserved**	**Sum**	***N***
**SYNTHETIC BIOLOGY (%)**							
Near future	19.0	4.5	34.4	18.6	23.6	100.0	695
Distant future	12.7	3.0	23.1	15.4	45.8	100.0	299
**GENOME EDITING (%)**							
Near future	12.8	7.6	32.7	16.7	30.2	100.0	695
Distant future	5.0	7.0	24.4	13.7	49.8	100.0	299

Interestingly, reservations in the response to emerging technologies depended on both the cognitive image of the self and the cognitive image of future generations, but the directions of impact were different between the two. Respondents who had a narrow self-concept were likely to postpone judgement, while those who set future generations in the distant future were likely to postpone judgement. A supplemental analysis revealed an inverse relationship between self-concept and opinion on future generations.

## Discussion

Indecision in public attitudes toward emerging biotechnologies is explicitly illustrated by our finding that around 80% of the respondents showed a two-sided, non-judgmental, or reserved attitude toward synthetic biology and genome editing. This indecision was found to be related to the cognitive image of the self and future generations. Our survey analysis demonstrated that those with a narrow cognitive self-concept tend to postpone judgement on emerging biotechnologies. Those with a broad cognitive self-concept tend to have a two-sided attitude and are more short-sighted but make judgments based on the impact of their decisions on the current and future generations.

It is important to examine more nuanced views when discussing the ethical, legal, and social implications of synthetic biology and genome editing. Our survey results show that those with a narrow cognitive self-concept tend to remain undecided about the application of emerging biotechnologies. This has two implications. First, it is important to understand the dynamics of their problem setting and framing for more enlightened and democratic policy making regarding synthetic biology and genome editing (Betten et al., 2018; Cavaliere et al., 2019). Second, narrow and broad cognitive self-concepts can be interpreted as reflecting two kinds of awareness, namely, episteme and phronesis. Those with a narrow cognitive self-concept follow an uncertain and contingent way of knowing (phronesis) and leave their decisions open before making a reasoned choice to adopt the best course of action (Fowers, 2003). Likewise, those who set future generations in the distant future may perform phronesis when they are undecided about biotechnology issues. Further studies should also examine how their attitude is affected by the human tendency to prefer smaller payoffs in the present over larger payoffs in future in the face of uncertainty (e.g., Dasgupta and Maskin, 2005).

Excluding a few studies (e.g., del Savio et al., 2015), the term “future generations” has not been clearly defined in the fields of economics, psychology, philosophy, and bioethics; rather, it has been used to generally indicate an indefinite future society (Parfit, 1982; Cooke, 2003; Wade-Benzoni et al., 2008; Arrow et al., 2013). However, when ethical, legal, and social implications are considered for the nature of emerging biotechnologies, the terms related to future can play a significant role in decision-making. This study revealed how people perceive the term “future generations” differently, indicating that different respondents made different decisions based on various scopes and ranges of future. These diverse and nuanced public responses are expected to effectively serve policy making in the complex, dynamic social systems in which new biotechnologies will emerge, as compared to aggregated responses by conventional public surveys.

Our findings should be applied to other social, cultural, and policy contexts with caution when the meaning of “future generations” and respondents' attitudes vary by language, country, and culture. For instance, East Asians, including the Japanese, are more likely to select more neutral, moderate, and ambivalent answers than North Americans (e.g., Chen et al., 1995). A comparative study will assess the effects of respondents' perceptions and recognitions across different countries and cultures. It would also be significant to examine the correlation between public response style and educational backgrounds. Together, all these endeavors offer elements in the crucial task of increasing the value of public questionnaires and encouraging “evidence-informed” policymaking (Head, 2015) on emerging biotechnologies.

## Data Availability Statement

The raw data supporting the conclusions of this manuscript will be made available by the authors, without undue reservation, to any qualified researcher.

## Ethics Statement

This paper presents data from an internet survey. Survey participants were comprised of panels developed by a Japanese research company. After being informed about the purposes of this study and the outline of questions, participants agreed to respond the survey. Completion of the questionnaire was regarded as consent to this study by participants. The right not to participate or to withdraw from this survey was guaranteed by not answering the survey. This study did not get written informed consent, according to the Code of Ethics and Conduct of the Japanese Psychological Association which does not require written informed consent from participants for anonymous surveys. Research Ethics Committee in the Faculty of Humanities and Social Sciences, Hirosaki University, approved the study in October 2017 (No. 2017-01) after reviewing all study materials.

## Author Contributions

AH, GY, and JM conceived and designed the study and acquired the data. AH analyzed the data and drafted the manuscript. GY and JM substantially revised the manuscript. All authors made a substantial contribution toward development of the final manuscript and approved publication.

### Conflict of Interest

The authors declare that the research was conducted in the absence of any commercial or financial relationships that could be construed as a potential conflict of interest.
